# Flexibility in metabolic rate and activity level determines individual variation in overwinter performance

**DOI:** 10.1007/s00442-016-3697-z

**Published:** 2016-07-26

**Authors:** Sonya K. Auer, Karine Salin, Graeme J. Anderson, Neil B. Metcalfe

**Affiliations:** Institute of Biodiversity, Animal Health and Comparative Medicine, Graham Kerr Building, University of Glasgow, Glasgow, G12 8QQ UK

**Keywords:** Activity rate, Intraspecific variation, Lipid stores, Metabolic rate, Phenotypic flexibility

## Abstract

Energy stores are essential for the overwinter survival of many temperate and polar animals, but individuals within a species often differ in how quickly they deplete their reserves. These disparities in overwinter performance may be explained by differences in their physiological and behavioral flexibility in response to food scarcity. However, little is known about whether individuals exhibit correlated or independent changes in these traits, and how these phenotypic changes collectively affect their winter energy use. We examined individual flexibility in both standard metabolic rate and activity level in response to food scarcity and their combined consequences for depletion of lipid stores among overwintering brown trout (*Salmo trutta*). Metabolism and activity tended to decrease, yet individuals exhibited striking differences in their physiological and behavioral flexibility. The rate of lipid depletion was negatively related to decreases in both metabolic and activity rates, with the smallest lipid loss over the simulated winter period occurring in individuals that had the greatest reductions in metabolism and/or activity. However, changes in metabolism and activity were negatively correlated; those individuals that decreased their SMR to a greater extent tended to increase their activity rates, and vice versa, suggesting among-individual variation in strategies for coping with food scarcity.

## Introduction

Many organisms live in seasonal environments where they must cope with fluctuations in food availability. Winter in temperate and polar regions can be particularly challenging because food is typically limited or non-existent during this time. As such, organisms must often rely on stored energy (Witter and Cuthill 1993; Berg and Bremset 1998; Næsje et al. 2006; Sheriff et al. 2013). However, depletion of these stores can have grave consequences, individuals with lower energy reserves being more prone to mortality through starvation (Haramis et al. 1986; Biro et al. 2004; Finstad et al. 2004; Krams et al. 2013), or greater exposure to predation through increased foraging activity (Bull et al. 1996). Given the strong selection pressure to retain energy stores above critical limits (Biro et al. 2004), mechanisms that mediate the energy balance of organisms should be of vital importance.

The downregulation of baseline metabolic requirements (standard and basal metabolism in ectotherms and endotherms, respectively) in response to food scarcity is common among many animal species (Hervant et al. 2001; Ostrowski et al. 2006; McCue 2007; Nord et al. 2009; Roark and Bjorndal 2009; Auer et al. 2015a), and occurs over timescales from days to weeks (Fu et al. 2005; Van Leeuwen et al. 2012). In addition, many animals reduce their activity levels to varying extents during the winter months, either by entering hibernation (O’Farrell 1974; Tanaka 2006) or by reducing their foraging rates (Gardiner and Geddes 1980; Cunjak and Power 1986; Cotton and Parker 2000). However, members of a population can vary considerably in how they respond to food scarcity: some individuals may decrease while others are less flexible or even increase their rates of metabolism (Fu et al. 2005; Auer et al. 2015a) or activity (Krause et al. 1998; Killen et al. 2011). This intraspecific variation in reversible phenotypic changes, or phenotypic flexibility (sensu Piersma and Drent 2003), of physiological and/or behavioral traits may, in turn, explain why some individuals deplete their energy reserves more quickly than others (Schultz and Conover 1999; Biro et al. 2004; McKenzie et al. 2014). However, little is known about whether individuals exhibit parallel or contrasting changes in their metabolic and activity rates, and how these phenotypic changes collectively affect their overwinter performance.

Links between metabolic rate and behavioral traits have been hypothesized, but their functional basis is currently under debate (Careau et al. 2008; Biro and Stamps 2010; Mathot and Dingemanse 2015). On the one hand, baseline metabolism represents the summed energetic expense of maintaining all the systems and functions critical for life, so it may also reflect the energetic capacity to perform behaviors, including activity (*performance model*; Careau et al. 2008; Biro and Stamps 2010). Thus, individuals may show positively correlated changes in baseline metabolism and activity rate in response to food scarcity. Alternatively, competition for limited energy may lead to an allocation-based trade-off between baseline metabolism and activity whereby energy diverted to one trait comes at a cost to the other (*allocation* model; Careau et al. 2008). Thus, individuals may maintain their current baseline metabolic rate but then have to reduce their activity when food levels decline, or they may maintain their current activity rates but then need to reduce their resting metabolism, leading to a negative correlation between changes in the two traits. Finally, there may not be any functional linkage between the two traits (*independent* model; Careau et al. 2008; Mathot and Dingemanse 2015). Thus, individuals may undergo independent, uncorrelated changes in their metabolism and activity rates.

The above models make distinct predictions for the covariance between rates of metabolism and activity, yet empirical evidence is thus far equivocal. Positive, negative, and nonsignificant intraspecific correlations have all been reported (Biro and Stamps 2010; Toscano and Monaco 2015). However, there is also increasing evidence that the direction and magnitude of the relationship between the two traits can change with environmental circumstances, being significant under some but not all conditions (Killen et al. 2013). For example, metabolic rate is positively correlated with rates of activity in European sea bass under the more stressful conditions of food deprivation or hypoxia but not when food or oxygen availability is high (Killen et al. 2011, 2012). These context-dependent links suggest that individuals may differ in the degree to which their metabolism relative to their activity level changes in response to food scarcity. However, intraspecific variation in the flexibility of both metabolic and activity rates has not yet been evaluated simultaneously. Thus, it is not clear whether and how these two traits covary and thus how they might together affect individual variation in overwinter performance.

We examined individual changes in both standard metabolic rate (SMR) and activity level and their consequences for rates of lipid depletion among juvenile brown trout (*Salmo trutta*) experiencing simulated winter conditions of cold temperatures and progressively decreasing rations. Brown trout are known to downregulate their standard metabolic rate when food levels decline (Auer et al. 2015a). Juvenile salmonid fishes are also known to decrease their activity levels during the winter time, spending the daytime hidden in streambed refuges (Metcalfe et al. 1999) where they feed little (Gardiner and Geddes 1980; Metcalfe and Thorpe 1992) and rely heavily on their lipid stores (Berg and Bremset 1998; Finstad et al. 2004). Minimizing the rate of depletion of these energy reserves is essential for their overwinter survival (Biro et al. 2004; Finstad et al. 2004), so an individual’s ability to downregulate its metabolism and/or reduce its activity rates may therefore be an important fitness determinant during this key time of the year.

## Materials and methods

### Fish origin and care

Trout (*n* = 25) were the offspring of wild-origin parents from the River Tweed, Scotland; the experimental fish were drawn at random from a stock population of 19 full-sib families. The fish had hatched over the 2013–2014 winter and been reared under conditions of ad libitum food in communal stock tanks under ambient temperature and 12L:12D light conditions in an indoor aquarium facility at the University of Glasgow. During December 2014, water temperature and light levels were set to constant winter conditions (7.5 °C and 8L:16D). In January 2015, after a month of acclimation to these ‘winter’ conditions, the fish were anaesthetized under a mild solution of benzocaine (40 mg/L), weighed (±0.001 g), and then transferred to individual compartments in a flow-through stream system in the same aquarium facility and under the same conditions of light and temperature. The individual compartments were each equipped with a small shelter (pottery chard) for the fish and were separated by a net partition that allowed fish to see one another but prevented the downstream movement of food (Auer et al. 2015b). Each fish was hand-fed twice daily with an individually calculated ration of commercial pellets (EWOS, Westfield, UK) based on an equation modified from Elliott (1976) that predicts the daily caloric intake of brown trout needed to produce a moderate growth rate for a fish of a given body mass (*W*) in grams and at a given temperature (*T*): calories = 14 *W*^0.737^*e*^0.128*T*^. The daily ration (mg trout pellets) for each fish was roughly 0.4 % body mass per day and was determined by converting the required daily caloric intake into trout pellets (mg) using published values of the energetic content of the trout pellets (EWOS, Westfield, UK).

### Experimental protocol

In February 2015, after fish had been on this ‘intermediate’ ration for 1 month, their standard metabolic rate and body mass were determined, as well as other biometric measures of fish shape and size (for calculation of initial lipid content, see below). At this time, fish body mass and length ranged from 3.76 to 7.79 g (mean ± 1 SE: 5.70 ± 0.24) and from 72.33 to 92.14 mm (mean ± 1 SE: 82.65 ± 1.16), respectively. They were returned to their stream compartments, and their ration, in calories, was reduced to 3.2 *W*^0.96^*e*^0.08*T*^. Fish were fed this ration once daily for the first 2 weeks, twice weekly for the third week, and then not at all for the final 2 weeks of the experiment. These rations corresponded to roughly 0.1, 0.001, and 0 % of their body mass on a daily basis, respectively, and were designed to reflect a steady seasonal decrease in food availability (and so avoid an unnatural abrupt cessation of food). The 5 week duration of the experiment was chosen because brown trout are known to alter their SMR over that time period in response to changing food availability (Auer et al. 2015a). As such, changes in activity levels, if any, were also expected to occur over that same time frame. Compartments were cleaned every 2–3 days to remove fecal matter and maintain water quality. Fish activity rates were recorded 1 week and then 5 weeks after their initial SMR measurements, corresponding to the first and last week of the 5-week food reduction period. Standard metabolic rate, body mass, and biometric measurements were determined again for each fish at the end of the 5 week period. Fish were then euthanized immediately, thereafter, with an overdose of benzocaine and frozen at −70 °C for analyses of lipid stores.

### Metabolic rates

Standard metabolic rate was measured as the rate of oxygen consumption using continuous flow-through respirometry (Auer et al. 2015b). The experimental setup consisted of 14 glass chambers arranged in parallel and submerged in water. Aerated water was pumped via oxygen-impermeable tubing from an upper bin to each respirometry chamber and then past an oxygen sensor to a lower bin before being recirculated back to the upper bin. A chiller and UV sterilizer connected in series were used to keep the water at a constant 7.5 °C and to minimize background respiration rates, respectively. Oxygen concentration of the water flowing out of the chambers was recorded every 2 s using multichannel oxygen meters and attached sensors and associated FireSting software (version 3.0, PyroScience, Aachen, Germany). The main bin containing the respirometry chambers was insulated and covered in a dark cloth to keep fish activity to a minimum.

Fish were in a postabsorptive state prior to each measurement of their metabolic rate (Rosenfeld et al. 2015)—ensured on the first occasion by not feeding them for 48 h prior to measurements. They were then placed in individual respirometry chambers and their oxygen consumption was measured over a 22 h period (from roughly 1200–1000 the following day). One chamber was fish free and served as a control measure of background respiration rates. Flow rates were set to 1.05 L h^−1^ to allow detection of oxygen consumption rates but not allow oxygen levels to drop below 80 % saturation. Standard metabolic rate (mg O_2_ h^−1^) was measured as *M*_O2_ = *V*_w_ × (*C*_wO2control_ − *C*_wO2fish_), where *V*_w_ is the flow rate of water through the respirometry chamber (L h^−1^), and *C*_wO2control_ and *C*_wO2fish_ are the concentrations of oxygen (mg L^−1^) in the outflow of the chambers lacking and containing fish, respectively. Fish were measured in a total of two batches over 2 days (12–13 fish per batch) for both initial and final measurements. On each occasion, the standard metabolic rate for a fish was calculated by taking the mean of the lowest 10^th^ percentile of oxygen consumption measurements over the 22 h measurement period, and then excluding outliers, i.e., those measurements below two standard deviations from this mean (Auer et al. 2015b).

### Activity rates

A digital camcorder (Panasonic HC-V700) was used to film the activity rates of fish in their individual compartments of the stream system over 3 h episodes. Filming took place 1 week after the first metabolic rate measurement and then during the fifth week just before the final metabolic rate measurement. During the first week, filming started 2–3 h after feeding. Fish divided their time between resting in various locations—inside, on top of, or beside the shelter—and swimming quickly from one of these resting locations to another. A fish’s activity rate, measured only for the final 2 h of each filming episode to remove any potential biases caused by human presence during initial video setup, was quantified as the frequency with which it moved between these resting locations (Killen et al. 2012). Activity rate was then expressed as the mean number of such movements per hour.

### Lipid content

Fish were thawed and placed in 60 ml glass bottles in a drying oven at 60 °C for 4 days. A 4 day drying period was chosen because pilot trials on a different set of fish (*n* = 10) showed that they reached a constant body mass (±0.01 g) within that time frame. Fish were removed from the oven, weighed (=dry mass), and then submerged in diethyl ether. The following day, the ether was replaced by a fresh solution and the fish left submerged overnight. After this second 24 h period of submersion, the ether was clear and colorless indicating that all the lipids had been extracted and the fish had reached a constant lean mass (Reznick 1983). This method extracts non-polar lipids, the mobilizable energy substrates that trout are known to rely on throughout the winter (Berg et al. 2011). Fish were then dried overnight in a drying oven and weighed the next day (=lean dry mass). The lipid mass for each fish was calculated as the difference between the dry mass and the lean dry mass (Auer et al. 2010).

The relationship between lipid stores and biometric measures of each fish at the end of the experiment was then used to estimate its fat content at the start of the experiment and thereby estimate the loss of its lipid stores over the food reduction period. Specifically, we first determined the relationship between lipid stores of fish and the following biometrics measured at the end of the experiment (linear measurements were recorded ±0.01 mm using digital calipers): their final body mass (M), fork length (L), adipose fin length (ADFL), body height (HO) and width (WO) taken just behind the operculum, body height (HD) and width (WD) taken at the point where the dorsal fin arises from the body, and body height (HA) and width (WA) taken at the point where the anal fin arises from the body (Simpson et al. 1992). Together, these biometric measures explained most of the variation in lipid stores (*R*^2^ = 0.90), so the equation relating lipid stores (g) to these biometric measures [lipid weight = 0.968 + 0.142 M − 0.024L + 0.012ADFL − 0.034HO + 0.037HD + 0.027HA − 0.008WO + 0.0001WD + 0.028WA] was then used together with those same biometric measures of the fish taken at the start of the experiment to predict the initial lipid content of each fish (based on methods described by Simpson et al. 1992). The specific, or proportional, loss of lipids over the experimental period was calculated for each fish as: 100 * [log_10_ (final lipid content) − log_10_ (initial lipid content)] (Jobling 1983).

### Statistical analyses

We first examined whether SMR changed over the experimental period. The model included log_10_-transformed SMR as the dependent variable, measurement time (initial vs. final) as a fixed categorical effect, log_10_-transformed body mass at the time of each SMR measurement as a continuous predictor, and individual identity as a random effect to account for the non-independence of repeated measurements. SMR at the start and end of the experimental period may have been affected by activity rates (ACT), as postulated by the allocation model, so ACT at each measurement time was included as a covariate. The same approach was used to test whether ACT changed over the experimental period while accounting for body mass and individual identity. Likewise, SMR at each measurement time was included as a covariate since it could have a positive or negative effect on ACT, as postulated by the performance and allocation models, respectively. We then examined whether there were consistent differences among individuals in their SMR and ACT across time, i.e., if their final SMR and ACT were positive functions of their initial SMR and ACT, respectively. We also tested separately whether the change in SMR (ΔSMR: final SMR − initial SMR) and the change in ACT (ΔACT: final ACT − initial ACT) were a function of initial SMR and ACT, after correcting for regression to the mean (Kelly and Price 2005). We then tested whether ΔSMR and ΔACT were correlated, after accounting for effects of initial SMR and ACT and regression to the mean (Kelly and Price 2005).

We then tested whether lipid stores decreased over the experimental period. The model included lipid stores (% wet mass) as the dependent variable, measurement time (initial vs. final) as a fixed categorical effect, and individual identity as a random effect. Finally, we examined whether a fish’s ΔSMR and ΔACT explained changes in its lipid stores (percent lipid loss, as defined above) while accounting for its initial SMR and ACT. Changes in lipid stores are known to be a function of a fish’s body size (Schultz and Conover 1999; Biro et al. 2004), so log_10_-transformed initial mass was also included as covariate. Changes in lipid stores could alternatively be related to the final SMR or ACT rather than ΔSMR or ΔACT, respectively. However, final SMR and ΔSMR were positively correlated (Pearson’s *r* = 0.69, *p* < 0.001) as were final ACT and ΔACT (Pearson’s *r* = 0.52, *p* < 0.001), so effects of ΔSMR and ΔACT were evaluated in a separate model from final SMR and ACT to avoid problems of collinearity.

SMR and activity were both functions of body mass, so mass-independent estimates, i.e., residuals of individual SMR (rSMR) and activity rates (rACT), were generated from their regression against body mass, standardised to a common mean body size of 5.5 g, and used in analyses of change where body mass was included as a covariate so as to avoid problems associated with their collinearity. Changes in SMR, ACT, and lipid stores may be a function of a fish’s body mass and/or lipid stores, so these variables were included as covariates in analyses of change, but subsequently removed when nonsignificant (*P* > 0.05). Batch number was included as a random effect in all analyses to account for the order in which fish entered the experiment. Analyses were conducted using SPSS version 22 (SPSS Inc., Chicago, IL, USA). Although percent lipid loss, metabolic and activity rates were modeled as continuous traits, we refer to their extremes in the Results and Discussion section simply to illustrate comparisons of individuals with contrasting responses. Effects were considered significant when *p* < 0.05. All means given are ±1SE.

## Results

### Changes in metabolic and activity rates

**Fig. 1:**
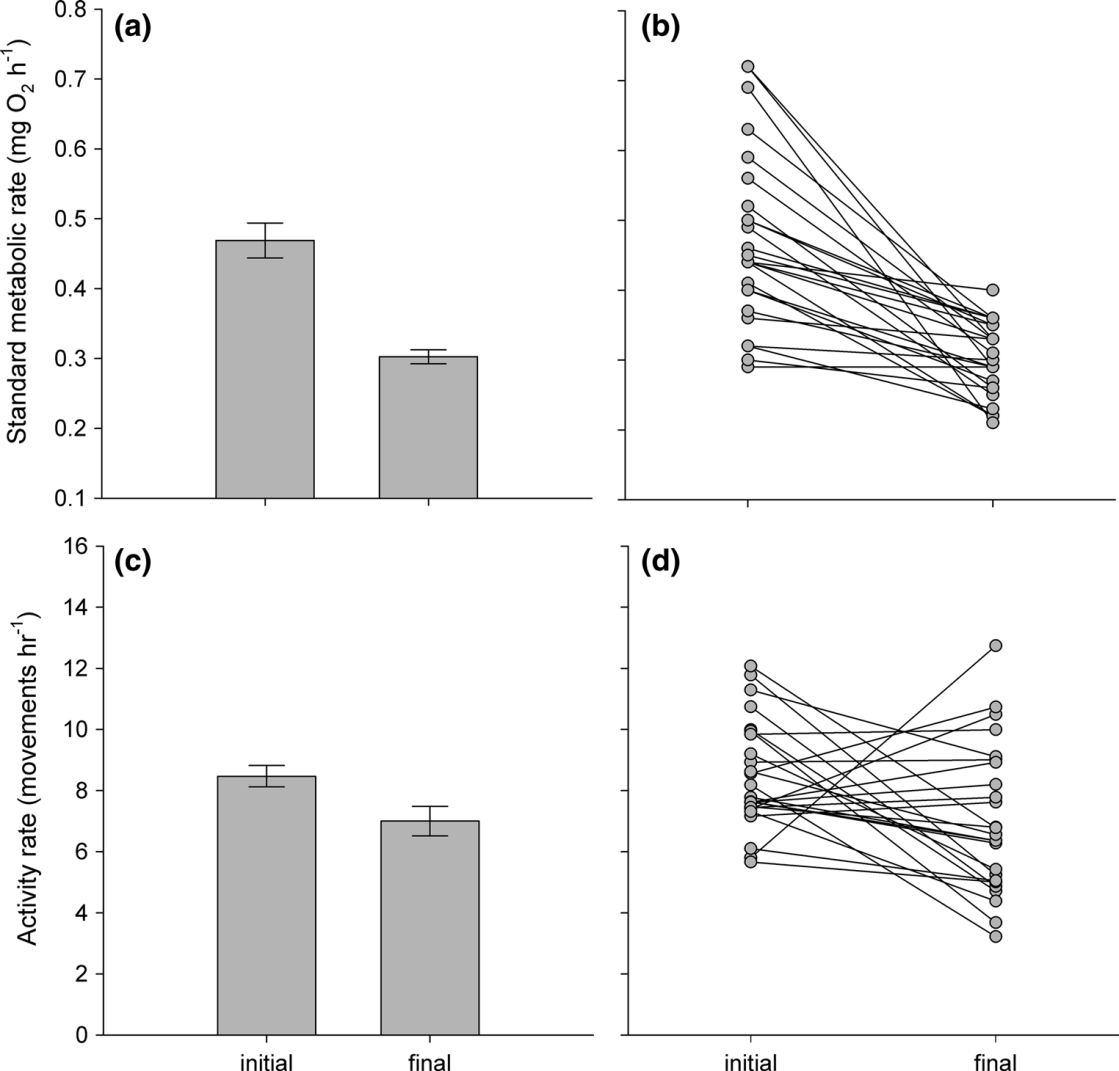
Mean and individual changes in standard metabolic rate (**a**, **b**), and activity rate (**c**, **d**) of juvenile brown trout (*Salmo trutta*, *n* = 25) over a 5 week period of decreasing rations at 7.5 °C. Plotted are partial residuals evaluated at the means of all other predictors in the models

Log_10_-transformed SMR decreased over the food reduction period (Fig. 1a; *t*_1,34.0_ = −3.36, *p* < 0.01) after controlling for the positive effects of log_10_-transformed body mass (1.54 ± 0.28; *t*_1,36.0_ = 5.52, *p* < 0.001) and the negative effects of log_10_-transformed lipid stores (−0.39 ± 0.14; *t*_1,25.8_ = −2.86, *p* < 0.01). In addition, rACT had a negative effect on SMR (*t*_1,30.9_ = −3.58, *p* < 0.01), but the magnitude of its effect did not differ significantly across measurement times (*t*_1,30.8_ = 0.98, *p* = 0.34). Mean activity rate also decreased over the food reduction period (Fig. 1c; *t*_1,26.6_ = −2.16, *p* = 0.04) after controlling for the negative effects of body mass (−0.75 ± 0.32; *t*_1,22.8_ = −2.36, *p* = 0.03). In addition, rSMR had a negative effect on ACT (*t*_1,44.4_ = −2.21, *p* = 0.03) that did not differ across measurement times (*t*_1,42.3_ = 1.44, *p* = 0.16). Individuals differed considerably in how their SMR and ACT changed over the experimental period (Fig. 1b, d), but their final rSMR and final rACT were not functions of their initial rSMR (*t*_1,23.0_ = 0.31, *p* = 0.76) or initial rACT (*t*_1,22.0_ = 0.55, *p* = 0.59), respectively.

### Covariance between metabolism and activity

ΔSMR was not a function of initial rSMR (*t*_1,20.0_ = 0.89, *p* = 0.38) or initial rACT (*t*_1, 20.0_ = −0.30, *p* = 0.77) but of initial body mass and lipid stores; individuals with a smaller initial log_10_-transformed body mass (1.43 ± 0.67; *t*_1, 20.0_ = 2.13, *p* = 0.04) and a larger initial lipid store (−1.22 ± 0.56; *t*_1, 20.0_ = −2.20, *p* = 0.04) reduced their SMR to a greater extent. Likewise, an individual’s ΔACT was not a function of its initial rSMR (*t*_1,22.0_ = −0.12, *p* = 0.91) or rACT (*t*_1,22.0_ = 0.15, *p* = 0.88).

**Fig. 2:**
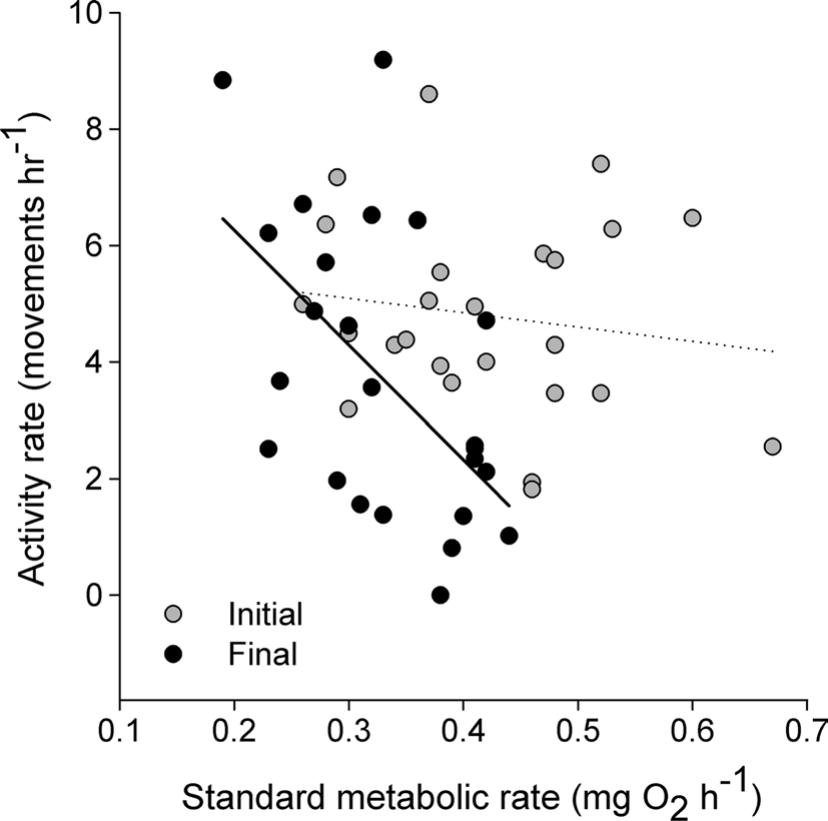
Correlation between activity and metabolic rates of juvenile brown trout (*Salmo trutta*, *n* = 25) at the start (initial: *r* = −0.16, *P* = 0.45) and end (final: *r* = −0.69, *p* < 0.01) of a 5 week period of decreasing rations at 7.5 °C. Values are standardized for the mean body mass of 5.5 g

**Fig. 3:**
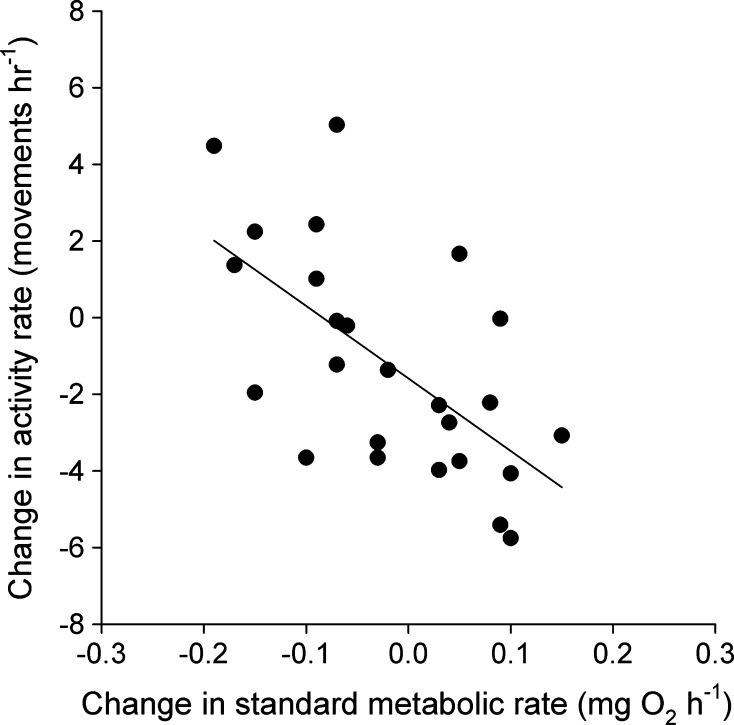
Correlation between changes in standard metabolic rate and activity rate (*r* = −0.63, *p* < 0.02) among individual juvenile brown trout (*Salmo trutta*, *n* = 25) over a 5 week period of decreasing rations at 7.5 °C. Plotted are partial residuals evaluated at the means of all other predictors in the model

While the relationship between SMR and ACT did not differ across measurement times (see above), their relationship among individuals went from being nonsignificant at the start (Fig. 2; *r* = −0.17, *p* = 0.45) to statistically significant and negative by the end of the experiment (Fig. 2; *r* = −0.69, *p* < 0.01). As such, ΔSMR was negatively correlated with ΔACT (*r* = −0.63, *p* < 0.01); individuals with the greatest decrease in their SMR tended to show no change and sometimes an increase in activity levels, and vice versa (Fig. 3).

### Consequences for lipid stores

**Fig. 4:**
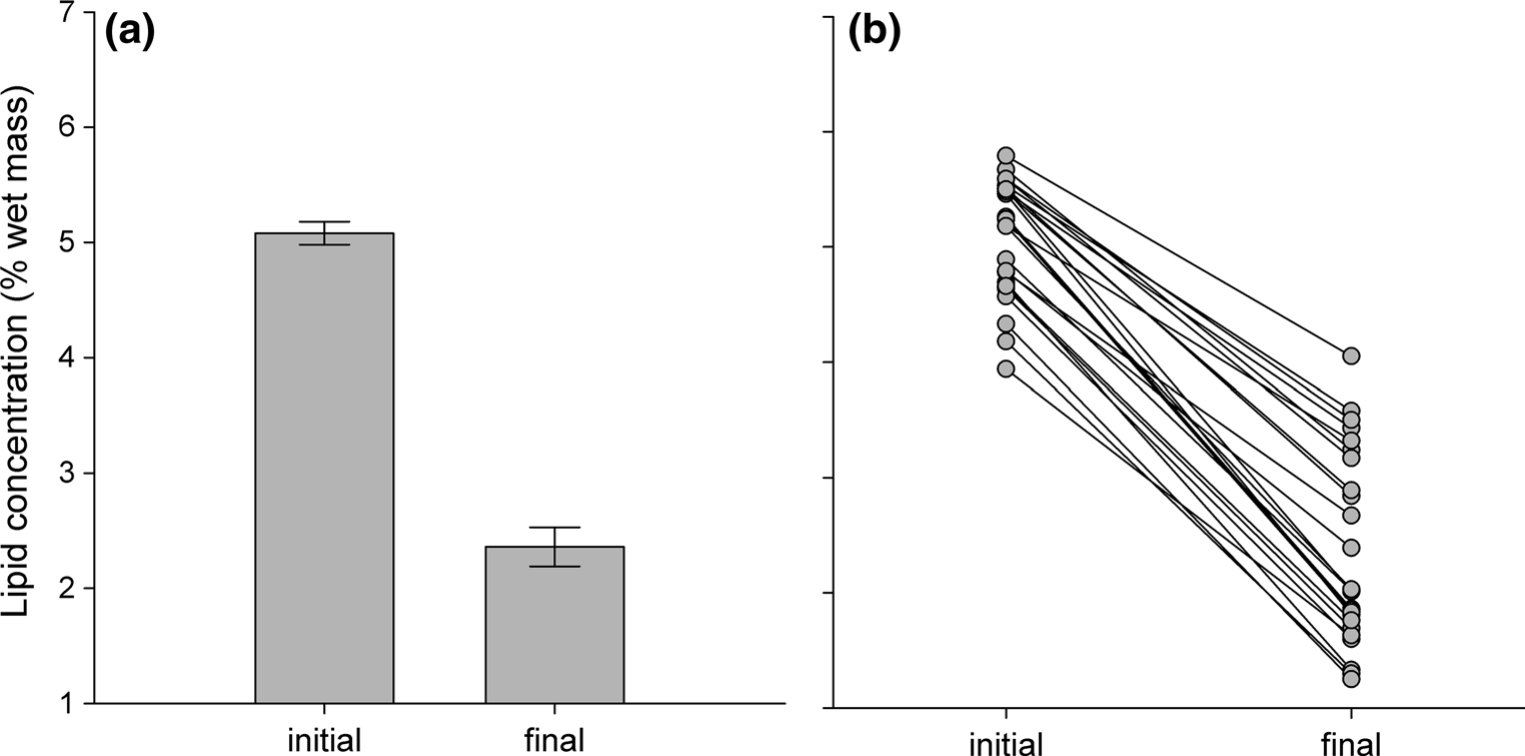
**a** Mean and **b** individual changes in lipid stores of juvenile brown trout (*Salmo trutta*, *n* = 25) over a 5 week period of decreasing rations at 7.5 °C

**Fig. 5:**
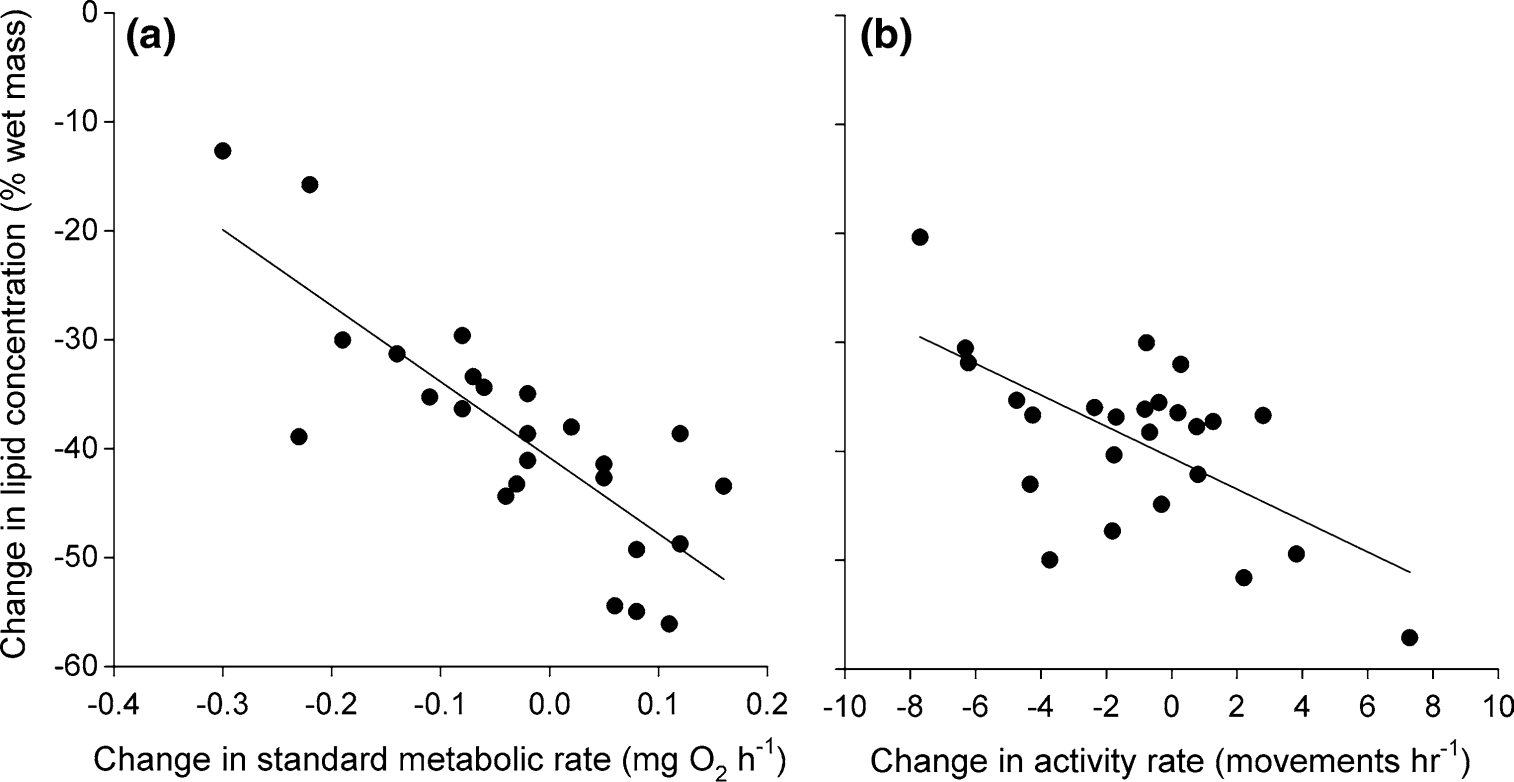
Change in lipid stores of individual juvenile brown trout (*Salmo trutta*, *n* = 25) as a function of the change in their **a** standard metabolic rate and **b** activity rate after 5 weeks of decreasing rations at 7.5 °C. Plotted are partial residuals evaluated at the means of all other predictors in the model

Estimated lipid stores (as a percentage of wet mass) decreased significantly over the 5 week experimental period (Fig. 4a; *t*_1,24_ = 23.9, *p* < 0.001), but individuals differed in their estimated percent loss (Fig. 4b). These individual differences in lipid depletion were explained by changes in metabolism and activity rates. Individuals with a larger initial log_10_-transformed body mass (97.25 ± 15.38, *t*_1,19.0_ = 6.32, *p* < 0.01), a greater decrease in rSMR (Fig. 5a; *t*_1, 19.0_ = −3.88, *p* < 0.01), a greater decrease in rACT (Fig. 5b; *t*_1, 19.0_ = −2.61, *p* = 0.01), and a lower initial rACT (−1.79 ± 0.84, *t*_1, 19.0_ = −2.13, *p* = 0.04) lost a smaller percentage of their lipid stores. However, percent lipid loss was not related to initial rSMR (*t*_1, 19.0_ = −1.84, *p* = 0.08). When final rSMR and rACT instead of ΔrSMR and ΔrSMR were included in the model, results were qualitatively the same except that initial rACT was no longer a significant predictor: individuals with a larger log_10_-transformed body mass (96.66 ± 15.35, *t*_1,19.0_ = 6.30, *p* < 0.01), a low final rSMR (−58.52 ± 15.06, *t*_1, 19.0_ = −3.89, *p* < 0.01), and a lower final rACT (−1.47 ± 0.55, *t*_1, 19.0_ = −2.67, *p* = 0.01) lost a smaller percentage of their lipid stores. However, these specific changes in lipid stores were not predicted by initial rSMR (*t*_1, 19.0_ = 1.50, *p* = 0.15) or initial rACT (*t*_1, 19.0_ = −0.50, *p* = 0.62).

## Discussion

Average rates of metabolism and activity decreased in response to food scarcity, yet individuals exhibited striking differences in their phenotypic flexibility. Interestingly, changes in metabolism and activity were negatively correlated; individuals that decreased their SMR to a greater extent did not change or even increased their activity rates and vice versa. These changes were significant predictors of patterns of lipid depletion; individuals that reduced both their SMR and ACT to a greater extent during the experiment, and therefore had a lower final SMR and ACT, depleted fewer lipid stores than those individuals that were less flexible and had a higher final SMR and/or activity. These differences in flexibility led to 3-fold variation among individuals in the minimum and maximum loss of lipids.

The rank order of an individual’s SMR and ACT, i.e., its SMR and ACT relative to others in the population, is generally consistent over time when measured under standardized conditions and/or short time intervals (Nespolo and Franco 2007; Biro and Stamps 2010; Auer et al. 2016). However, there is increasing evidence that both SMR and ACT can change in response to food availability (McKechnie 2008; Killen et al. 2011; Auer et al. 2015a) as well as other factors such as temperature (McKechnie et al. 2007; Biro et al. 2010) and oxygen concentration (Hochachka et al. 1996; Killen et al. 2012). In addition, it is not well known whether an individual’s SMR and ACT in a given environment can predict its SMR and ACT if conditions change. Here, we found that individuals did not exhibit consistent differences in their metabolic and activity rates as food availability decreased over the 5 week period. This finding corroborates that reported for activity levels of another fish species measured under varying levels of water-oxygen concentrations (Killen et al. 2012) and for basal metabolic rates of a bird species measured across consecutive winters (Cortes et al. 2015). However, it differs from the higher degree of repeatability of SMR found in a salamander species under changing temperatures (Careau et al. 2014). These contrasting results may arise due to a number of factors including differences in the type or magnitude of environmental change, responses of different species or traits to environmental variation, or variation in the interval duration between measurements. While the covariation between physiological and behavioral traits across changing environments remains poorly studied, these results demonstrate at the very least that individuals do not always exhibit temporally consistent differences in their physiological and behavioral traits in variable environments.

Changes in metabolism and activity were not functions of initial SMR or ACT. Rather, they were affected by one another and in a negative direction. This negative relationship is consistent with predictions from the allocation model that posits a trade-off among the two traits due to their common reliance on a limited energy source (Careau et al. 2008). However, individuals on the whole did not exhibit simple binary responses in which they decreased their energy allocation to one trait but not the other. At the extremes, some individuals reduced their SMR but not their ACT, while others reduced their ACT but not their SMR, but many more exhibited intermediate changes in both traits. These differences in phenotypic flexibility, in turn, led to a change in the statistical significance of the intraspecific association between SMR and ACT; while the correlation between the two traits did not differ between measurements times, their relationship was statistically different from zero only under conditions of food deprivation and not when food was more available at the start of the experiment. These results demonstrate that differences among individuals in their sensitivity to environmental change can influence the direction and magnitude of the association between physiological and behavioral traits at the among-individual level and thereby explain, at least in part, why correlations can differ among studies and environmental conditions (Killen et al. 2013).

Estimated decreases in lipid stores are similar to those reported in semi-natural and field studies of overwintering brown trout and other salmonid fishes (Gardiner and Geddes 1980; Berg and Bremset 1998). Yet, there was considerable variation around this average, with some individuals suffering twice the lipid loss of others. These differences among individuals were explained by variation in phenotypic flexibility, but why changes in metabolism and activity differed both within and among individuals is unclear. The downregulation of SMR in response to declining food availability is facilitated by changes in processes such as digestion and assimilation (Armstrong and Bond 2013), mitochondrial efficiency (Monternier et al. 2014), and/or respiratory substrate use (McCue 2010). Differences in these underlying functions may therefore explain why some individuals can respond more to a decline in food availability than others. However, while many individuals decreased their SMR and ACT, others did not change or even increased their SMR and/or ACT despite the associated decrease in their lipid stores to levels considered near to the minimum concentration needed for survival (roughly 1 % wet mass; Biro et al. 2004). These contrasting responses in metabolism and activity levels among individuals strongly suggest that intraspecific variation in flexibility may be a consequence not just of functional constraints but also of restraint (Auld et al. 2009). That is, there may be limits but also costs to flexibility that must be weighed against the benefits of maintaining energy reserves. With respect to metabolism, reductions in the masses of organs such as those associated with digestion are a cost-effective means of coping with decreases in food availability (Barboza et al. 2010; Armstrong and Bond 2013), but may be disadvantageous if their upregulation delays an individual’s ability to exploit resources once conditions improve (Biebach 1998). Additionally, any further decrease in minimal energy expenditure may come at a cost to somatic maintenance and repair needed for future survival. With respect to activity, individuals trying to manage their energy reserves over the winter face a double-edged sword: if they feed little, they may deplete their energy reserves and die of starvation, but if they attempt to forage to try and replenish their energy reserves, their increased activity rates require additional energy and may also increase their risk of predation in the wild (McNamara and Houston 1990; Brodin 2007). Individual variation in metabolic and behavioral flexibility may therefore reflect alternative strategies for coping with food scarcity.

Changes in lipid stores were a function of the change in mass-independent SMR and ACT but also of initial body size, larger individuals losing a lower percentage of their lipids than smaller ones despite exhibiting smaller reductions in their SMR. This finding mirrors size-dependent patterns reported in other species (Lindstedt and Boyce 1985; Krause et al. 1998; Schultz and Conover 1999; Biro et al. 2004). The lower levels of lipid depletion in larger individuals are thought to be a consequence of their lower mass-specific metabolism (Schultz and Conover 1999; Biro et al. 2004). However, activity rates also varied as a function of size, larger individuals being more sedentary than smaller ones. As such, size-differences in lipid depletion may be driven by the energy demands of both metabolism and activity rates that, in turn, may help explain why smaller individuals often have higher overwinter mortality (Loison et al. 1999; Biro et al. 2004).

We suggest, here, that flexibility in standard metabolic rate and activity levels may be important mechanisms for maintaining energy stores during periods of low food availability. Flexibility in these traits likely plays an important role in allowing organisms to cope with a diversity of environmental challenges. However, individuals can vary considerably in their phenotypic flexibility in both of these traits, and our understanding of the causes and consequences of this intraspecific variation is still in a nascent stage. Given the current global scale of human alteration to both habitat and climate, there is an urgent need to quantify individual capacities for change and whether populations as a whole will be able to adapt to or cope with these new and changing environments. Standard metabolic rate and activity levels are fundamental traits underlying organismal performance (Biro and Stamps 2010; Mathot and Dingemanse 2015), so further study is needed to assess the costs, benefits, and limitations of their flexibility in the face of this rapid environmental change.
